# Global Resistance of Imipenem/Relebactam against Gram-Negative Bacilli: Systematic Review and Meta-Analysis

**DOI:** 10.1016/j.curtheres.2023.100723

**Published:** 2023-10-28

**Authors:** Reza Abniki, Amirhossein Tashakor, Melika Masoudi, Davood Mansury

**Affiliations:** 1 Student Research Committee, Isfahan University of Medical Sciences, Isfahan, Iran; 2 Department of Microbiology, School of Medicine, Isfahan University of Medical Sciences, Isfahan, Iran

**Keywords:** antibiotic resistance, gram-negative bacilli, imipenem/relebactam

## Abstract

**Background:**

Relebactam, previously known as MK-7655, is currently being tested in combination with imipenem as a class A and class C β-lactamase inhibitor, including KPC from *Klebsiella pneumoniae*.

**Objective:**

The objective of the current study was to evaluate the activity of imipenem/relebactam against gram-negative bacilli.

**Methods:**

After applying exclusion and inclusion criteria, 72 articles with full texts that describe the prevalence of imipenem/relebactam resistance were chosen for the meta-analysis and systematic review. Articles published between January 2015 and February 2023 were surveyed. The systematic literature search was conducted in PubMed, Web of Science, Google Scholar, and Scopus.

**Results:**

The pooled estimation of 282,621 sample isolates revealed that the prevalence rate of imipenem/relebactam resistance is roughly 14.6% (95% CI, 0.116%–0.182%).

**Conclusions:**

The findings of this analysis show that imipenem/relebactam resistance is rare in the majority of developed countries. Given that relebactam has proven to restore the activity of imipenem against current clinical isolates, further research into imipenem/relebactam is necessary.

## Introduction

Antibiotic resistance is among the biggest threats to human health, according to the World Health Organization.^1^ The occurrence of antibiotic resistance can be due to natural circumstances, but the biggest reason behind this issue is the misuse of these substances. This has led to increased morbidity, mortality, and higher health care costs. Among these costs is the cost associated with treatment of multidrug-resistant (MDR) bacteria.^2^ As a result, using carbapenems such as imipenem has become quite popular against MDR bacteria. Carbapenems, just like the rest of the β-lactams, prevent the formation of bacterial cell walls and by this route they can kill these microorganisms.^3^ Excessive use of carbapenems has resulted in the occurrence of carbapenem resistance in various gram-negative bacteria families, including *Enterobacterales* and other bacteria (eg, *Pseudomonas aeruginosa* and *Acinetobacter baumannii*).^4^

Carbapenem-resistant *Enterobacterales* are considered a highly critical group of MDR organisms according to the World Health Organization.^5^ *Enterobacterales* (eg, *Klebsiella* spp, *Escherichia coli*, and *Enterobacter* spp) can cause both community- and health care-associated infections, and carbapenems can be considered among the last resources for treatment of organisms that produce extended-spectrum β-lactamase and AmpC.^6^^,^^7^ Misuse of carbapenems has led to an increase in the number of carbapenem-resistant *Enterobacterales* bacteria that produce carbapenemase enzymes.^8^ Other carbapenem-resistant species (eg, carbapenem-resistant *P aeruginosa* and carbapenem-resistant *A baumannii*) can induce hospital-acquired infections, which can lead to high mortality and morbidity, especially among critically ill patients. Physicians use various mechanisms to combat this resistance (eg, efflux pumps and enzymes) and eliminate MDR pathogens, but not too many antimicrobial molecules exhibit in vitro activity, therefore reducing antimicrobial therapy options.^9^^,^^10^

**Figure 1:**
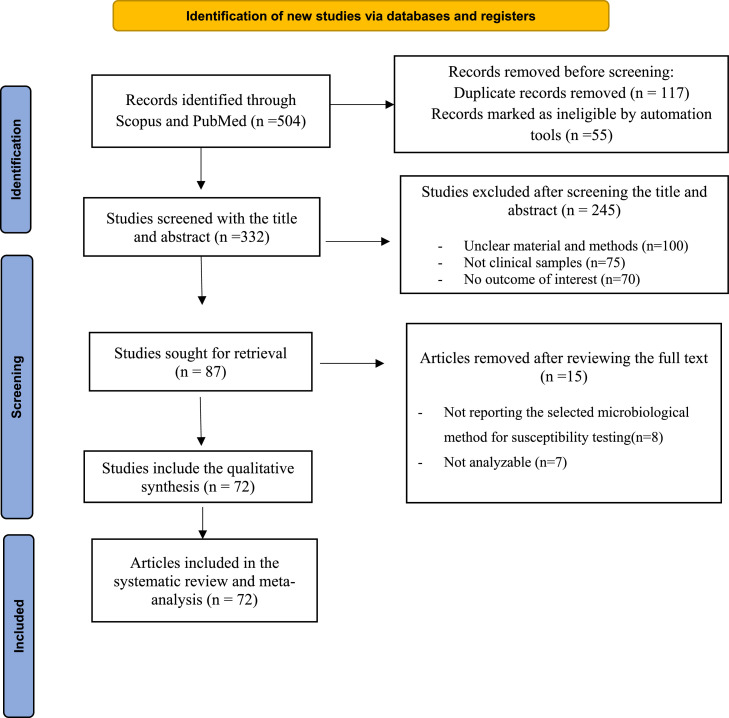
Flowchart of the literature searches strategy and study selection.

To limit carbapenem-resistant bacteria, different β-lactam–β-lactamase inhibitor combinations have been developed, 1 of which is imipenem/relebactam (IMI/REL). Relebactam is a β-lactamase inhibitor that, when combined with imipenem and cilastatin shows promising activity against certain carbapenem-resistant bacteria.^11^ Unfortunately, according to various studies, there is an emerging resistance toward this new combination even as it is being developed.^11^^,^^12^ In addition, its use has been reported in different countries around the world.^13^^,^^14^ The aim of this study is to assess the prevalence of IMI/REL resistance in gram-negative bacilli around the world (Figure 1).

### Selection criteria

This literature review and meta-analysis is based on systematic searches of multiple literature databases, including Medline/PubMed, Web of Science, Wiley Online Library, and the Cochrane library. Additional searches were performed in Scopus and Google Scholar. The following key words were used: (((*imipenem, cilastatin*, and *relebactam*) AND *relebactam*) AND *Drug Resistance*) AND *Gram-Negative Bacteria* [Mesh].

In accordance with the inclusion criteria, the title, abstract, and full texts were independently reviewed by 2 reviewers who also checked the search results for relevant database key words. Also, all articles from January 2015 to February 2023 were gathered. Consensus was used to settle disagreements among the reviewers. Duplicates were excluded when exporting the search results to Endnote. EndNote's headquarters is located at 20 Adolph Tidemands Gate, Lillestrom, Akershus, 2000, Norway. Clarivate is the sole and exclusive owner of all rights in the EndNote product. The choice of articles could be made in any language. Persian studies couldn't be located. Inclusion criteria are listed below:

• Antimicrobial activity was determined using standard methods such as broth microdilution and agar dilution and disk diffusion,
• The MIC and MIC range was reported, and
• Studies with clinical isolates were accepted.

At the same time, articles that did not use standard antimicrobial susceptibility methods or were <10 isolates per species and animal or environmental isolates were excluded.

### Quality of the evidence

Two authors independently assessed the quality of eligible studies using the Checklist for Improving the Reporting of Observational Studies in Epidemiology.^15^ Each item was judged on its suitability for the study type, sample size, population, inclusion/exclusion criteria for the original study, analytic methods, and results presentation.

### Data extraction

The first author's name, study duration, publication date, research location, antimicrobial susceptibility testing strategy, sample size, bacterial species, MIC results, and characteristics of antibiotic resistance were all taken from the articles that were chosen.

### Statistical Analysis

The meta-analysis was executed employing the Comprehensive Meta-Analysis Software version 3 (Biostat Inc, Englewood, New Jersey). SEs were calculated using a 95% CI, and for determining statistical significance, a *P* value of 0.05 was used. Regardless of heterogeneity, the analysis was conducted using a random-effects model. *I*^2^ statistics were used to determine the degree of heterogeneity; heterogeneity below 40 was regarded as low, between 40 and 60 as moderate, and above 60 as high. A funnel plot and the Egger rank correlation method were employed to evaluate potential publication bias. Two-tailed *P* < 0.05 was considered indicative of a significant publication bias. The relative weight for each study was also calculated.

## Results

### Literature search

For this study, a total of 504 articles were assessed. One hundred seventy-two articles were initially disqualified based on title evaluation because 117 of them were duplicate publications of the same study and 55 of them had records that automation tools had flagged as ineligible. Following the initial title and abstract screening, 245 articles were eliminated. Fifteen studies were ultimately excluded after full-text analysis because they did not contain the qualitative synthesis. All the articles published up until October 2022 were evaluated for this systematic review, and there was no time limit taken into consideration for including these articles in the study. Ultimately, 72 articles were chosen and used in the analysis (Figure 7,Table 1).

**Table 1:** Studies included in the meta-analysis.

First author	Publication year	Country	Sample type (bacteria)	Sample size	Resistance, %	Phenotype method	Reference No.
López-Pérezetal	2021	Spain	*Pseudomonas aeruginosa*	40	27.50	MicroScan WalkAway system (Beckman)	^36^
Mashaly	2021	Egypt	*Klebsiella pneumoniae*	140	54.30	BMD	^37^
Rybak	2021	USA	Gram-negative bacteria	21	11	BMD	^38^
Hernández-Garcíaetal	2022	Spain	*Enterobacterales* isolates	747	3.60	BMD	^39^
Yang	2021	Taiwan	*Escherichia coli, K pneumoniae*	660	16.20	Agar dilution test	^13^
Castanheira	2021	United States	carbapenemase-negative carbapenem-resistant *Enterobacterales*	45	11.10	BMD	^40^
Kurihara	2022	Japan	*Klebsiella aerogenes, Serratia marcescens*	61	19.6	Broth microdilution method and agar dilution method	^41^
Sader	2021	United States	*P aeruginosa*	360	1.70	BMD	^42^
Rolston	2022	United States	Gram-negative bacilli	440	5.20	BMD	^43^
Haines	2022	Australia	*P aeruginosa*	20	75	BMD	^44^
Danjean	2022	France	*Enterobacterales*	40	55	BMD	^45^
Karlowsky	2022	Canada	*P aeruginosa*	1050	22.30	BMD	^46^
Castanheira	2022	United States	*Enterobacterales*	450	38.10	BMD	^47^
Sader	2021	United States	*P aeruginosa*	583	5.70	BMD	^48^
Tamma	2022	United States	Carbapenem-resistant *Enterobacterales*	603	16	BMD	^49^
Lob	2020	United States	Enterobacteriaceae and *P aeruginosa*	19,870	1.90	BMD	^50^
Young	2019	United States	*P aeruginosa*	14813	10	BMD	^51^
Burgess	2019	United States	Carbapenem-resistant Enterobacteriaceae	96	0	BMD	^23^
Poirel	2022	Switzerland	*P aeruginosa* and *E coli*	70	21.4	BMD	^52^
Kuo	2021	Taiwan	imipenem-nonsusceptible *E coli, Kpneumoniae, Acinetobacter baumannii*, and *P aeruginosa*	397	48.10	BMD	^53^
Lob	2021	United States	*P aeruginosa*	1634	7.70	BMD	^54^
Tamma	2021	United States	*P aeruginosa*	32	81	BMD	^33^
Vázquez-Ucha	2021	Spain	Carbapenemase-producing *Enterobacterales*	401	14.20	BMD	^55^
Shields	2021	United States	*P aeruginosa*	32	37	BMD	^34^
Lob	2020	United States	Gram-negativ*e* bacilli	93565	3.96	BMD	^56^
Kuo	2020	Taiwan	*Elizabethkingia* spp	108	100	BMD	^57^
Nordmann	2021	Switzerland and Germany	*E coli*	33	100	BMD	^58^
Livermore	2020	United Kingdom	*P aeruginosa*	174	82	Agar dilution	^59^
Zhang	2021	China	*P aeruginosa* and *A baumannii*	3775	44	BMD	^60^
Karlowsky	2020	United States	*Enterobacterales, Paeruginosa, Stenotrophomonas maltophilia* and *Burkholderia* spp,	1,385	32	BMD	^61^
Bhagwat	2020	India	*E coli*	89	65.17	BMD	^62^
Yang	2020	China	Enterobacteriaceae	8781	4.80	BMD	^63^
Hakvoort	2020	United States	*Enterobacterales* and *P aeruginosa*	297	23.20	BMD	^64^
Nelson	2020	United States	Carbapenem-resistant *Enterobacterales*	598	20.80	BMD	^65^
Karlowsky	2019	United States	Non-proteeae Enterobacteriaceae and *P aeruginosa*	7517	0.80	BMD	^66^
Johnson	2020	United States	*E coli*	203	11	BMD	^67^
Fraile-Ribot	2020	Spain	*P aeruginosa*	1445	2.60	BMD	^68^
Karlowsky	2019	United States	Nonproteeae Enterobacteriaceae and *P aeruginosa*	1936	4	BMD	^69^
Carpenter	2019	United States	Carbapenemase-producing Enterobacteriaceae	200	94	BMD	^70^
Karlowsky	2020	United States	Enterobacteriaceae and *P aeruginosa*	13,248	4.90	BMD	^71^
Lob	2019	United States, Switzerland	*P aeruginosa*	5447	7.70	BMD	^72^
Galani	2019	Greece	*K pneumoniae*	314	7.30	BMD	^73^
Senchyna	2019	United States	Carbapenem-resistant Enterobacteriaceae	62	29	BMD and disk diffusion	^74^
Canver	2019	United States	Carbapenem-resistant *Enterobacteriaceae*	106	5	BMD	^75^
Asempa	2019	United States	Carbapenem-nonsusceptible *P aeruginosa*		8.50	BMD	^76^
Karlowsky	2018	United States	*P aeruginosa*	12,170	9.20	BMD	^77^
Gomez-Simmonds	2018	United States	*Enterobacteriaceae*	154	42.80	BMD	^78^
Schmidt-Malan	2018	United States, Canada, Singapore	Drug-resistant gram-negative bacilli	282	54	BMD	^79^
Karlowsky	2018	United States	*K pneumoniae, A baumannii, P aeruginosa,* and *Enterobacter* spp	4554	14.20	BMD	^80^
Karlowsky	2018	United States	Enterobacteriaceae and *P aeruginosa*	4037	1.90	BMD	^81^
Haidar	2017	United States	Carbapenem-resistant Enterobacteriaceae	100	12	BMD	^82^
Lob	2017	United States	*K pneumoniae, A baumannii, P aeruginosa*, and *Enterobacter* spp	2005	4.7	BMD	^83^
Lob	2017	United States	Nonproteeae Enterobacteriaceae, *P aeruginosa*	1451	4.50	BMD	^84^
Snydman	2016	United States	*Bacteroides* species	451	0.70	Agar dilution	^32^
Hernández-García	2022	Spain	Carbapenemase-producing *Enterobacterales*, carbapenem-resistant *Pseudomonas* spp	417	42	BMD	^85^
Horwich-Scholefield	2021	United States	Carbapenem-resistant *Enterobacterales*	75	20	BMD	^86^
Becka	2021	United States	*Burkholderia cepacia* complex, *Burkholderia gladioli*	150	28.60	BMD	^87^
Bail	2021	Brazil	*Enterobacterales, P aeruginosa*	529	18.3	BMD	^88^
Zhang	2022	United States	*P aeruginosa*	2531	8.50	BMD	^89^
Zhang	2022	United States	Carbapenem-nonsusceptible *Enterobacterales*	104	8.70	BMD	^90^
Walkty	2022	United States	*K pneumoniae*	375	9.10	BMD	^91^
Lob	2018	United States	Nonproteeae Enterobacteriaceae, *P aeruginosa*	56,533	4.20	BMD	^35^
Andrew Walkty	2021	Canada	*Enterobacterales, P aeruginosa*	5584	3.80	BMD	^92^
Lob	2021	United States	*Enterobacterales, P aeruginosa*	2640	0.90	BMD	^93^
MarakI	2021	Greece	*K pneumoniae*	40	100	Etest method	^94^
Bonnin	2022	France	OXA-48-like-producing *Enterobacterales*	362	18.70	BMD	^95^
Biagi	2022	United States	Metallo-b-lactamase-producing *enterobacterales*	13	100	BMD	^31^
Xu	2022	China	Carbapenem-resistant gram-negative pathogens	100	40	Agar dilution method	^14^
Papp-Wallace	2018	United States	*K pneumoniae* carbapenemase-producing Enterobacteriaceae	101	0	Agar dilution methods	^24^
Lapuebla	2015	United States	*E coli, K pneumoniae, Enterobacter* spp, *P aeruginosa*, and *A baumannii*	4697	3.30	Agar dilution method	^96^
Hernández-García	2022	Portuguese and Spain	*P aeruginosa*	474	6.30	BMD	^97^
Maraki	2021	Greece	Carbapenemase-producing *K pneumoniae*	266	24.80	MIC test strip method	^98^

BMD = broth microdilution method.

Our analysis's pooled estimation of 282,621 sample isolates revealed that the prevalence rate of IMI/REL resistance is roughly 14.6%. High levels of heterogeneity were observed (Cochrane *Q* test *P* = 0.0 and *I*^2^ = 99.53). In addition, we looked at the prevalence of imipenem resistance based on the sampling year of the studies that were included. By 2022, the resistance rate rose to 20.2% from 15.2% in 2020 (Figure 3).

**Figure 2:**
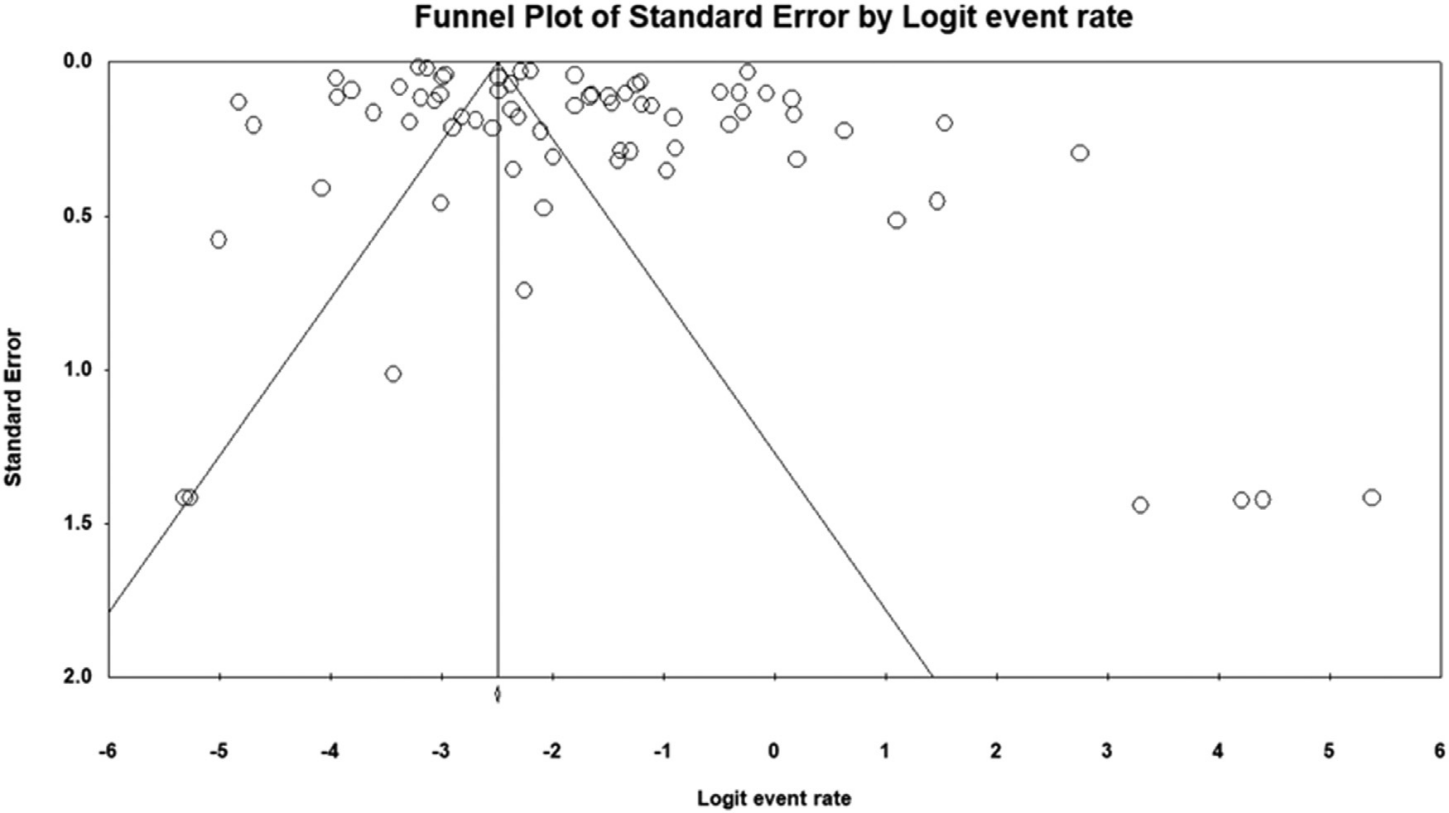
Funnel plot showing publication bias.

**Figure 3:**
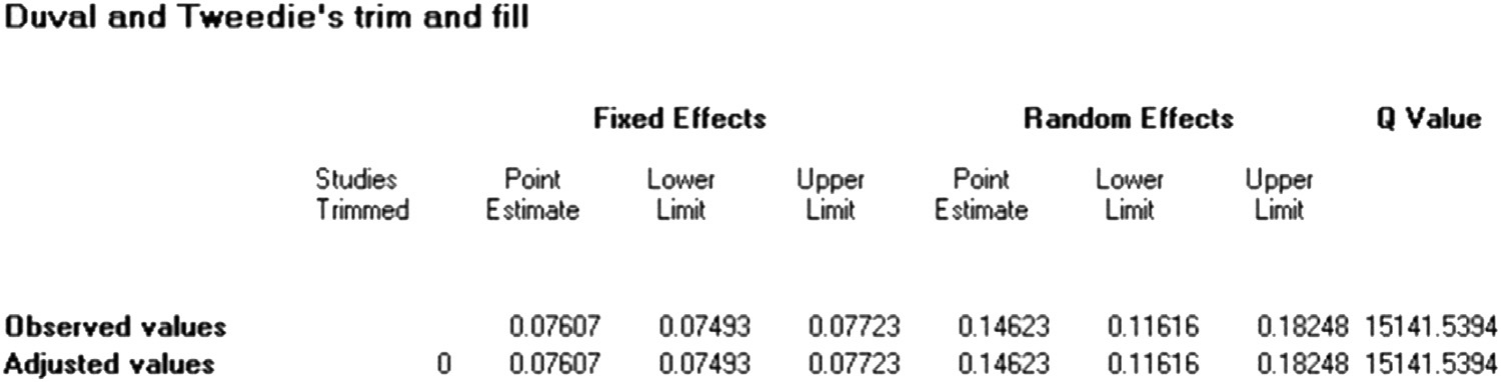
The fill method tries to estimate what is missing, whereas the trim method removes what is considered unreliable or extreme.

None of the studies that were included, according to the sensitivity analysis, significantly changed the overall resistance results. There was a slight asymmetry in the funnel plot. Asymmetry test showed a little evidence of publication bias that we will discuss further (Egger's test *P* = 0.004–0.009, Begg's test *P* = 0.407–0.819).

The included studies were carried out in 15 different countries. Most (44 studies) were conducted in the United States, 15 in Europe, 8 in Asia, 3 in Canada, 1 in Brazil, and 1 in Egypt. In all 72 articles, samples were of gram-negative bacilli. In 34 articles, *P aeruginosa* had been isolated from clinical samples. On the other hand, *Enterobacterales* was found in 52 studies, 33 which of were related to Enterobacteriaceae family (eg, *Klebsiella* spp, *E coli*, and *Serratia marcescens*). Other surveys were performed on other bacteria (eg, *Bacteroides* spp, *Burkholderia* spp, and *Acinetobacter* spp) (Table 1). Included studies were published between 2015 and 2022. The included studies mainly used the broth microdilution method for susceptibility testing, However, other techniques were agar dilution test, the E-test method, the MIC test strip method, MicroScan WalkAway system (Beckman) and disk diffusion test. The MicroScan WalkAway system, produced by Beckman Coulter. Brea, California, United States. The number of isolates investigated (sample sizes) in the studies varied from 13 to 93,565. The range of IMI/REL resistance in gram-negative bacteria are shown in Table 1.

### Publication bias

As we mentioned before, this systemic review and meta-analysis covers all of the studies performed around the world about resistance of gram-negative bacilli to IMI/REL. It is worth noting that the study encompasses a diverse range of sample sizes and varying rates of resistance. Table 2 includes the studies that were likely to be the reason for our publication bias (Figures 2 and 7).

**Table 2:** Studies with high risk of bias.

First author	Bacteria	Sample size	Resistance rate, %	*P* value	Weight
Uhlemann AC^78^	Enterobacteriaceae	154	42.8	0.077	1.50
Patel R^79^	Drug-resistant gram-negative bacilli	297	23.2	0.191	1.52
Mashaly ME^37^	*Klebsiella pneumoniae*	140	54.3	0.311	1.50
Kuo S-C^53^	*Elizabethkingia* spp	108	100	0.452	1.52
Haines RR^44^	*Pseudomonas aeruginosa*	20	75	0.033	1.26
Wenzler E^31^	Metallo-b-lactamase-producing *Enterobacterales*	13	100	0.022	0.56
Zhou T^14^	Carbapenem-resistant gram-Negative pathogens	100	40	0.047	1.49

## Discussion

Since the 1980s, there has been an alarming rise in the prevalence of MDR bacteria isolated from clinical samples. This fact calls for active surveillance of trends in antibiotic resistance due to widespread use and abuse of antibiotics.^1^^,^^16^^,^^17^ Because almost 80% of all antibiotics used in the health system are prescribed in primary care, this contribution is particularly significant.^18^

Bacteria strains that produce carbapenems have emerged as a result of the increased use of these drugs. Hospital infections are complicated by *Enterobacterales* that produce class A carbapenemase, such as *K pneumoniae* carbapenemase (KPC). IMI/REL is a novel antibiotic combination that inhibits the binding of penicillin-binding proteins (PBP1 and PBP2) to kill bacteria. It was recently (June 2020) given US Food and Drug Administration approval for use in hospital-acquired pneumonia and ventilator-associated pneumonia.^19^^,^^20^ Relebactam is a chemical compound used with imipenem to take role as a β-lactamase inhibitor and it blocks the ability of bacteria to destroy the β-lactam ring.^21^ Unfortunately, several studies have shown a concerning uprising in resistance toward this antibiotic resistance.^12^^,^^13^^,^^22^

**Figure 4:**
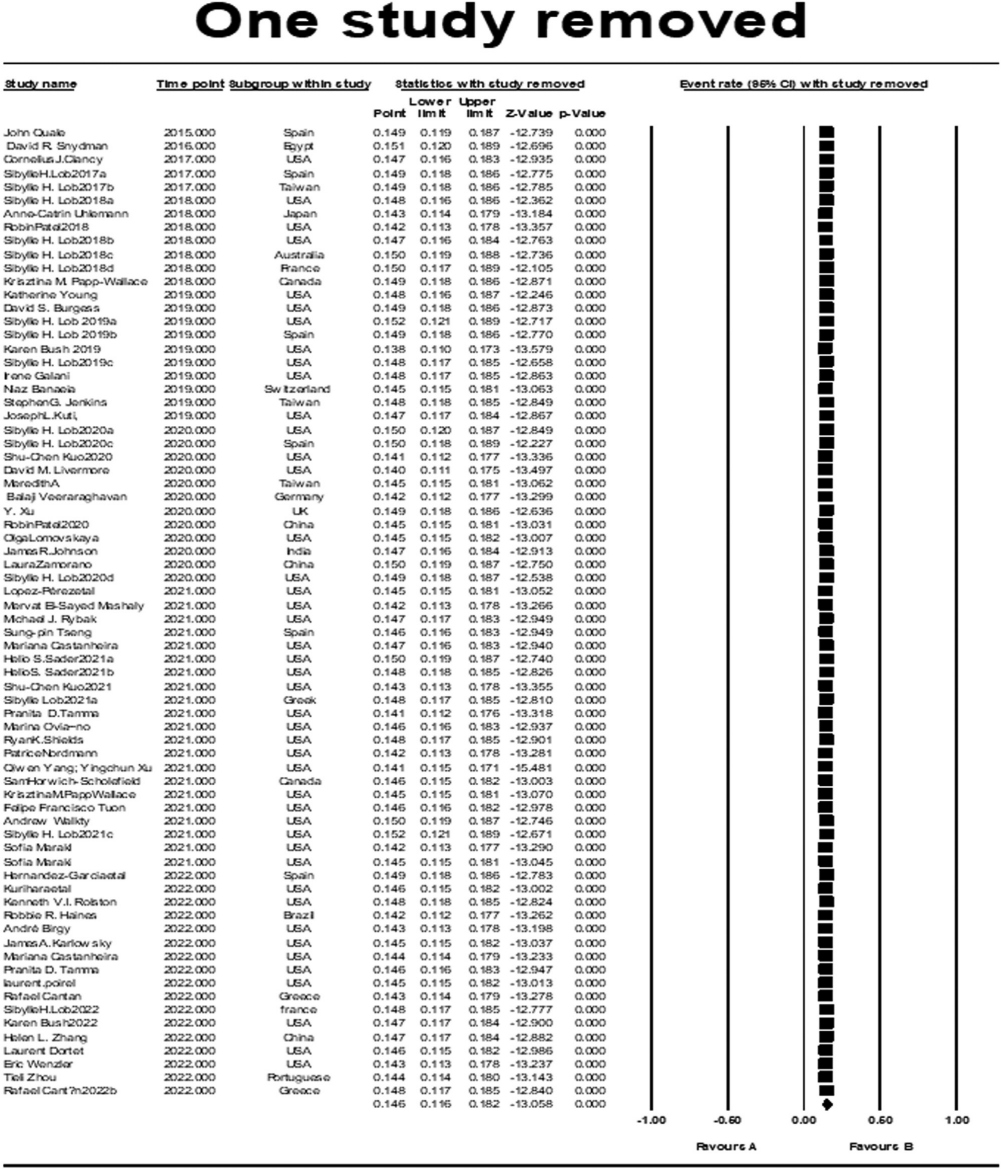
One-study-removed analysis.

**Figure 5:**
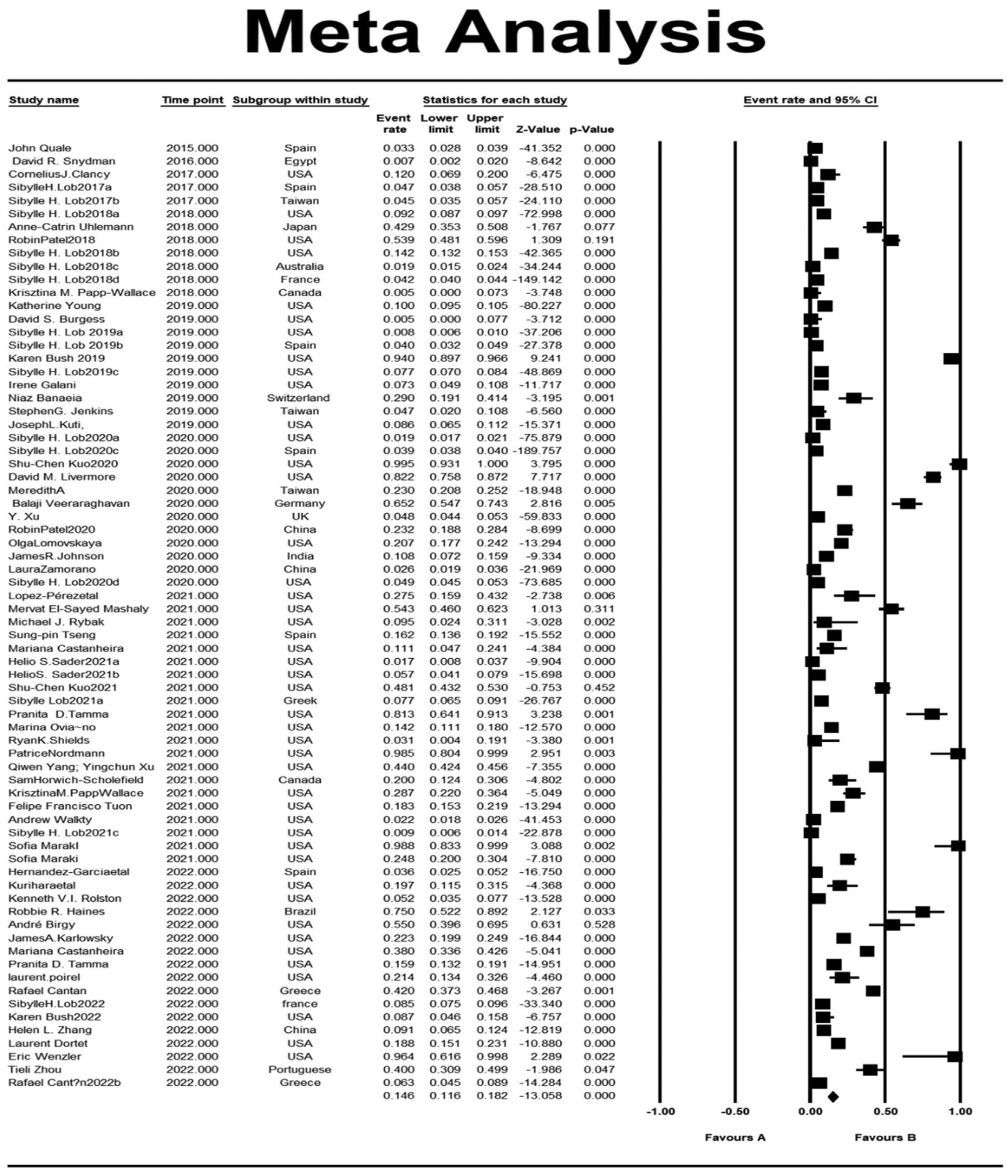
Forest plot depicting the overall event rate for the prevalence of imipenem/relebactam resistant isolates.

**Figure 6:**
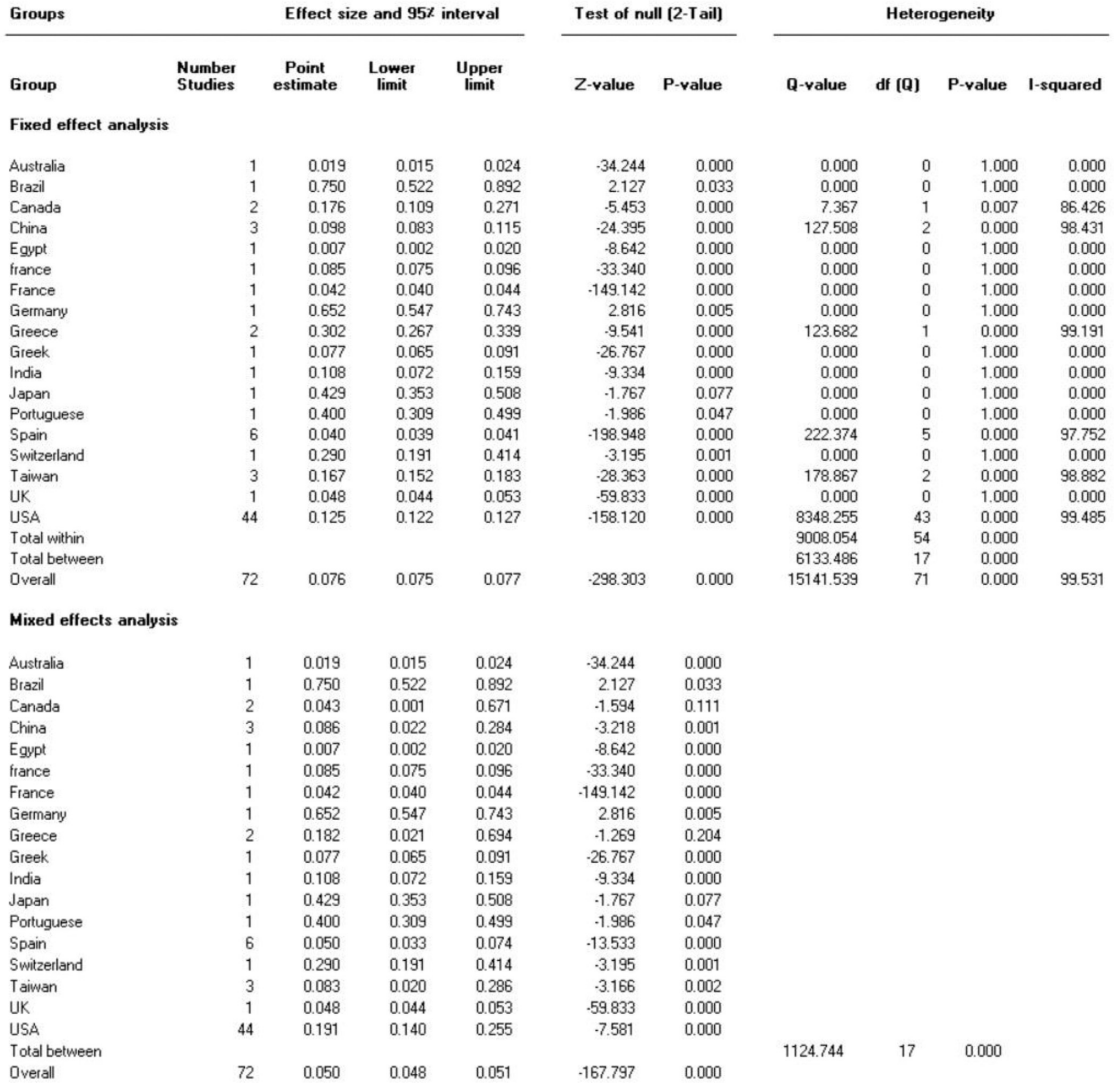
Subgroup analyses based on country.

We conducted the present systematic review and meta-analysis to estimate the cumulative prevalence of IMI/REL resistance in gram-negative bacilli by using data from 72 studies on various samples from the general population of 15 countries (Figure 6). There was a wide variety of sample sites such as blood, wound, urine, colostomy swab, respiratory, sputum, skin and soft tissue infections from patients residing on intensive care and nonintensive care units, severe immunocompromised adults, hospitalized patients with pneumonia, and so on. According to our meta-analysis, the prevalence of resistance was 14.6% and <50% in many countries around the world (Figures 4 and 5).

**Figure 7:**
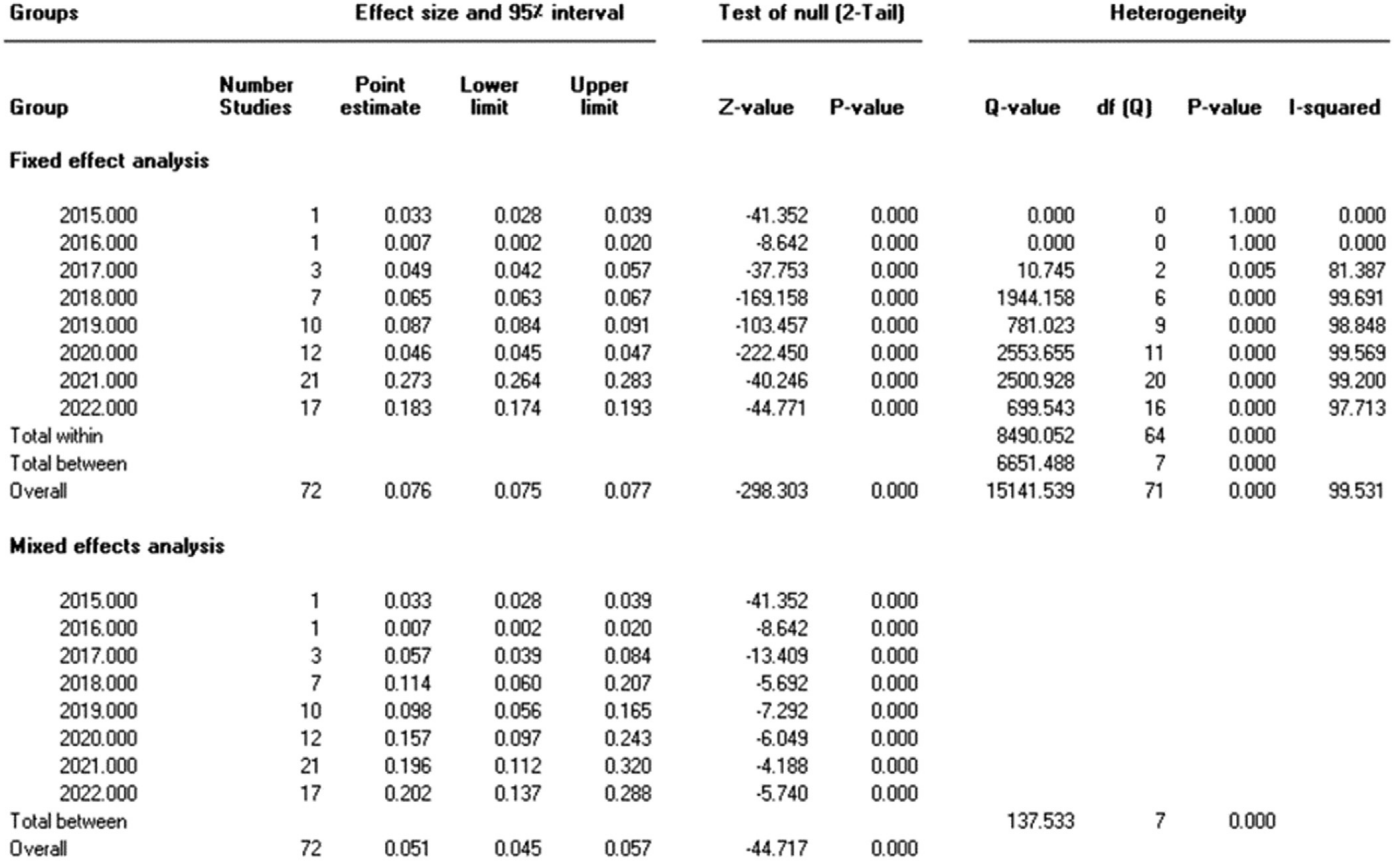
Association between sampling year and heterogeneity.

The broth microdilution method was used in a majority of the included articles for susceptibility testing, but other techniques such as agar dilution test, the E-test method, the MIC test strip method, the MicroScan WalkAway system, and the disk diffusion test were also performed.

Our findings also revealed that the lowest percentage of resistance was reported from the United States (0%) in 2 studies. Kulengowski and Burgess^23^ collected 96 arbapenemase-producing Enterobacteriaceae (CPE) clinical isolates from 2012 to 2017 in an academic medical center in Kentucky. They showed that isolates was 100% susceptible to IMI/REL, the distribution of IMI/REL MICs was around 0.25 to 1 mg/L, which is just under the imipenem susceptibility breakpoint of 1 mg/L (according to the 2018 Clinical and Laboratory Standards Institute guidelines). Another study conducted by Papp-Wallace et al^24^ surveyed that KPC-producing Enterobacteriaceae were 100% susceptible to an IMI/REL compound, with MICs ≤2 mg/L. Furthermore, the in vitro efficacy of IMI/REL against *P aeruginosa* strains lacking carbapenemases is significantly enhanced, exhibiting up to a 5-fold increase compared with imipenem alone. This enhanced activity extends to *P aeruginosa* strains possessing AmpC type β-lactamases associated with impermeability, as well as those producing KPC enzymes. Notably, the inclusion of relebactam resulted in a substantial shift in data, with the proportion of *P aeruginosa* strains resistant to carbapenems (ie, noncarbapenemase producers) and susceptible to imipenem decreasing from 2% to 63% upon exposure to IMI/REL.^25^

On the other hand, in 4 studies we found 100% resistance to this antibiotic. Hsu et al^26^ identified 108 clinical *Elizabethkingia* spp isolates. These microorganisms are gram-negative bacilli that do not ferment glucose and are aerobic, nonmotile, and are not spore forming. Three of the species are newly discovered opportunistic pathogens that can infect people seriously and are especially dangerous for those with compromised immune systems.^27^ *Elizabethkingia* spp exhibit high-level MDR. A variety of antibiotics have been tested in vitro.^28^, ^29^, ^30^ In the aforementioned study, *E anophelis, E meningoseptica*, and *E miricola* were totally resistant to IMI/REL with MIC >16.4. In elderly (ie, older than age 65 years) patients, the majority of isolates were found in blood or respiratory specimens. The samples came from patients who had been treated not only in intensive care units but also in wards and outpatient clinics at regional hospitals and clinics located throughout Taiwan. A study by Snydman et al^31^ with 13 clinical *Escherichia coli and K pneumonia* isolates, showed no susceptibility to IMI/REL with Etests result of 8, 16, 32, and 64 mg/L. By the way, that study could be 1 reason of our publication bias due to low the sample size (13 isolates) and *P* = 0.022.

In an article by Simner et al,^32^ almost all of the *Bacteroides* species were susceptible to IMI/REL (0.70% resistance). A comparison of the antimicrobial agents IMI/REL and imipenem alone against all of the isolates in this study revealed identical MIC_90_ values of 1 g/mL. Resistance was 0% for both imipenem and IMI/REL. Relebactam does not inhibit the metalloenzyme (cfiA gene) produced by the *Bacteroides fragilis* group, as evidenced by the lack of enhanced activity of the combination. Additionally, other resistance mechanisms, such as a porin mutation, may also be to blame for resistance to imipenem and other carbapenems.

Based on our research, MDR rates for Enterobacteriaceae, and difficult-to-treat resistance (DTR) and MDR rates for *P aeruginosa* were significantly higher (*P* < 0.05) in isolates collected in intensive care units than nonintensive care wards and in respiratory tract isolates than intra-abdominal or urinary tract isolates. Due to the Clinical and Laboratory Standards Institute breakpoint for susceptibility in *P aeruginosa* in the broth microdilution method (MIC) is 8.4 mg/L. The current study showed that 2 different studies that investigated IMI/REL resistance in *P aeruginosa* showed different resistance rates, although they had almost similar sample sizes. In 1 of them, which was a cohort of 32 paired DTR *P aeruginosa* isolates from 16 patients with an 81% (*P* = 0.001) resistance rate.^33^ Another study with same sample size (23 baseline and 32 postexposure MDR *P aeruginosa* isolates collected from 23 patients) showed 37% resistant to IMI/REL.^34^ On the flipside, we had a study with 10,834 *P aeruginosa* isolates from patients residing in intensive care or nonintensive care wards with 4.2% resistant to this antibiotic. Furthermore, according to the authors, IMI/REL had an activity rate of >90% against *P aeruginosa* in intensive care wards in the United States, Canada, the South Pacific, and Europe, compared with >82% in all other regions. Generally speaking, isolates from Respiratory tract infection (RTIs) were more resistant than those from Intra Abdominal Infection (IAIs), particularly among *P aeruginosa* and in isolates from nonintensive care settings.^35^ European countries such as France, Germany, Switzerland, Portugal, and Greece reported prevalence of resistant isolates below 20%.

Our findings support the use of high-quality in vitro studies to identify potentially effective combination regimens for use in clinical practice and to direct the choice of therapies in the future. When interpreting the findings, certain restrictions need to be taken into account. First, distinct phenotypic approaches should be considered because they might lead to varying reports on IMI/REL resistance. Second, due to the lack of pertinent data in many countries, our findings do not accurately reflect the resistance rate for all nations.

## Conclusions

The findings of this analysis underscore the rarity of IMI/REL resistance within the majority of developed nations. Consequently, policymakers and health care professionals must prioritize the implementation of more effective antibiotic stewardship and infection control strategies to mitigate the potential dissemination of IMI/REL resistance. Moreover, it is imperative that medical care centers, including hospitals and universities, across various regions of the world, such as Asia, Africa, Australia, Central Europe, and others, undertake comprehensive surveys to investigate the resistance patterns of multidrug bacteria toward IMI/REL. This rigorous approach is essential to ensure the acquisition of significantly more precise and reliable data. This study also emphasized the necessity of setting up reference labs with uniform standards for antimicrobial resistance surveillance to continuously monitor antibiotic resistance profile changes.

## Declaration of Competing Interest

The authors have indicated that they have no conflicts of interest regarding the content of this article.
